# Race, Structural Racism, and Prevalence of Diabetes in US Neighborhoods

**DOI:** 10.1001/jamanetworkopen.2026.5122

**Published:** 2026-04-06

**Authors:** Leonard E. Egede, Rebekah J. Walker, Jennifer A. Campbell, Obinna Ekwunife, Sebastian Linde

**Affiliations:** 1 Division of Population Health Department of Medicine, Jacobs School of Medicine and Biomedical Sciences, University at Buffalo, Buffalo, New York; 2 Department of Health Policy and Management, School of Public Health at Texas A&M University, College Station

## Abstract

**Importance:**

Race and measures of structural racism are associated with higher prevalence of diabetes at the census-tract level, but pathways and targets for intervention are unclear.

**Objective:**

To investigate the direct and indirect associations of race and 2 measures of structural racism (historic and contemporary) with prevalence of diabetes at the census-tract level.

**Design, Setting, and Participants:**

Cross-sectional study of 15 190 US census tracts across 157 counties within 50 states and Washington, DC conducted September 2025 to February 2026.

**Exposures:**

Historic structural racism was defined as historic redlining using the Home Owners’ Loan Corporation neighborhood grades (score between 1 = best and 4 = redlined) overlayed on present-day census tracts. Contemporary structural racism was defined using the structural racism effect index (SREI), a summary score of 9 domains (built environment, criminal justice, education, employment, housing, income and/or poverty, social cohesion, transportation, and wealth). African American or Black race was defined as proportion within a census tract based on American Community Survey 2019 data.

**Main Outcomes and Measures:**

Diabetes prevalence from the US Centers for Disease Control and Prevention Population Level Analysis and Community Estimates 2019 dataset was the main outcome. Structural equation modeling was used to investigate direct and indirect associations between race and both measures of structural racism. Analyses were completed using standardized estimates (β), which are interpreted as the change in SD associated with 1 SD increase in the variable.

**Results:**

Among 15 190 census tracts, mean (SD) diabetes prevalence across census tracts was 11.8% (4.9%), and a mean (SD) of 26.7% (31.4%) of the population within census tracts was African American or Black. Both historic (β = 0.02; 95% CI, −0.05 to 0.08; *P* = .001) and contemporary (β = 0.56; 95% CI, 0.51 to 0.61; *P* < .001) measures of structural racism were directly associated with higher diabetes prevalence. Historic structural racism was indirectly associated with higher diabetes prevalence via the pathway of contemporary structural racism (β = 0.17; 95% CI, 0.15 to 0.20; *P* < .001). Contemporary structural racism showed a stronger association compared with historic structural racism for the pathway through which African American or Black race was associated with higher diabetes prevalence (β = 0.58; 95% CI, 0.52 to 0.63 vs β = 0.18; 95% CI, 0.14 to 0.22; both *P* < .001).

**Conclusions and Relevance:**

African American or Black race, historic structural racism, and contemporary structural racism were associated with higher diabetes prevalence at the census-tract level in the US. Domains of contemporary structural racism as measured by the SREI are potential targets for future interventions.

## Key Points

**Question:**

Are race and 2 measures of structural racism (historic and contemporary) associated with the prevalence of diabetes at the US census-tract level, and what are the direct and indirect pathways for this association?

**Findings:**

In this cross-sectional study of 15 190 census tracts, there was a direct association between African American or Black race, historic structural racism and contemporary structural racism, and higher prevalence of diabetes at the census-tract level. In addition, there was an indirect association between African American or Black race and higher prevalence of diabetes at the census-tract level via contemporary structural racism.

**Meaning:**

These findings suggest that African American or Black race, historic structural racism, and contemporary structural racism were associated with higher diabetes prevalence at the census-tract level in the US.

## Teaser

This cross-sectional study evaluates direct and indirect associations between race, historic and contemporary measures of structural racism, and prevalence of diabetes at the census-tract level.

## Introduction

Diabetes remains a persistent and growing public health challenge, currently ranking as the seventh leading cause of death in the US^1^ and affecting 15.8% of the population.^2,3^ Diabetes also imposes a considerable economic burden on both individuals and society at large with an estimated cost of $412.9 billion annually in the US.^4^ Despite advances in prevention and management, disparities in diabetes prevalence, outcomes, and complications persist, particularly among African American or Black populations.^5,6^

Over the past decade, evidence shows the important role of social determinants of health in shaping health disparities. More recently, attention has shifted to upstream structural factors, such as systemic inequities in income, education, health care access, and neighborhood conditions, that shape the broader social environment and exacerbate diabetes-related outcomes.^7,8,9,10,11^ Structural racism, defined as the ways in which societies foster racial discrimination through mutually reinforcing systems, is a specific type of structural inequity linked to racial and ethnic disparities in health outcomes.^7,8,12,13,14,15,16,17,18^ To quantify and study its influence, researchers have developed a range of measures that capture structural racism across various levels and domains.^12,14^ One widely used historical measure is residential redlining, which reflects how discriminatory housing and lending practices contributed to persistent racial segregation and neighborhood-level disparities in wealth and opportunity.^19^ Historic residential redlining is often operationalized through scores developed by the Home Owner Loan Corporation (HOLC), which were used to determine access to credit and mortgage lending primarily in African American neighborhoods.^19^ More recently, contemporary structural racism has been operationalized through the Structural Racism Effect Index (SREI), which generates a composite score across 9 domains of influence based on 42 publicly available census-tract level variables.^20^

A recent systematic review examining the relationship between structural racism and diabetes outcomes found significant associations with poorer clinical indicators such as elevated HbA_1c_ and blood pressure, as well as lower adherence to self-care behaviors, individuals receiving less than the diabetes standards of care, higher mortality, and more years of life lost among adults with diabetes.^7^ The review also identified multiple pathways through which structural racism affects diabetes prevalence, including influences on incarceration, poverty, discrimination, substance use, housing, education, unemployment, and food access.^7^

While these findings underscore the broad and enduring influence of structural racism, less is known about how to develop interventions that address the relationship between race, structural racism, and prevalence of diabetes at the census-tract level. Therefore, the objective of this study was to investigate the association between race and 2 measures of structural racism (historic and contemporary) with the prevalence of diabetes at the census-tract level and identify direct and indirect pathways for this association. We hypothesized that (1) both historic and contemporary measures of structural racism would be directly associated with higher diabetes prevalence; (2) contemporary structural racism would serve as an indirect pathway through which historic residential redlining is associated with diabetes prevalence; and (3) both historic and contemporary racism would serve as indirect pathways through which African American or Black race is associated with higher diabetes prevalence.

## Methods

### Data Source and Study Population

This was a cross sectional study of data collected at the census-tract level. The analytic dataset was created by combining census tract–level data across multiple sources including the US Centers for Disease Control and Prevention (CDC) Population Level Analysis and Community Estimates (PLACES),^2^ Mapping Inequality Project,^21^ American Community Survey,^22^ and SREI data from Dyer et al.^20^ The sample population was all census tracts with data from both the Mapping Inequality Project to allow measurement of historic residential redlining and from the SREI to allow measurement of contemporary structural racism. The final sample consisted of 15 190 US census tracts across 157 counties, within 50 states and Washington, DC. Because the data source is publicly available and the data are anonymous, the study was exempt from institutional review board approval and the need for informed consent, in accordance with 45 CFR §46. The study followed Strengthening the Reporting of Observational Studies in Epidemiology (STROBE) reporting guidelines.

### Outcome Measure

Diabetes prevalence from the CDC PLACES 2019 dataset was used as the outcome of interest.^2^ CDC PLACES data are constructed from small-area measures using census tract–level model-based estimates derived from the Behavioral Risk Factor Surveillance System survey and the American Community Survey data.^2^ Multilevel regression and poststratification is used to compute probability of adults who report being told by a health care professional they have diabetes.^2^ The probability is applied to population estimates from the Census 2020 population counts or annual county-level estimates to generate prevalence.^2^

### Race, Historic Residential Redlining, and Contemporary Structural Racism

African American or Black race was defined using data from the American Community Survey. The proportion of the census tract reporting African American or Black race was used.

Historic redlining was defined using the HOLC scores for each census tract from the Mapping Inequality Project’s digitized HOLC security risk maps.^21^ The value of 1 indicates census tracts that are perfectly contained within historically graded A (or best) areas; a value of 2 for census tracts fully contained within historically graded B (or still desirable) areas; a value of 3 for census tracts fully contained within historically graded C (or definitely declining) areas; and a value of 4 for census tracts fully contained within historically graded D (or hazardous), or fully redlined areas. Census tracts that spanned multiple historic grading areas were given a value based on a weighted average of each area covered (ie, 50% covered within HOLC B and 50% within HOLC C would score 2.5).^8,23^ Therefore, scores ranged from 1 to 4 with higher scores indicating more of the census tract was redlined.

Contemporary structural racism was defined using the SREI, a summary score of 9 domains (built environment, criminal justice, education, employment, housing, income and/or poverty, social cohesion, transportation, and wealth).^20^ SREI was created using multiple sources of data across each domain.^20^ Examples of variables included in the index by domain include: built environment (vacancy rate, internet access, and low food access), criminal justice (jail rate and law enforcement personnel per capita), education (high school diploma, bachelor’s degree, and per pupil spending), employment (unemployment rate and retail job availability), housing (units without telephone, units without plumbing, crowding, foreclosure risk, and eviction risk), income and/or poverty (rate below poverty line, public assistance, family income, and per capita income), social cohesion (changed address in last year and income gap), transportation (carpool to work, access to vehicle, and public transport to work), and wealth (home value, median real estate taxes, median gross rent, and owner-occupied homes). The SREI score was validated by examining correlation with measures of health and other commonly used area-based indices, including the Area Deprivation Index, Social Vulnerability Index, and Social Deprivation Index.^20^ The SREI was consistently more strongly associated with health measures such as life expectancy compared with other indices.^20^ Scores have a mean of 0 and SD of 1, with negative scores indicating richer resources and positive scores indicating poorer resources.^20^

### Statistical Analyses

Structural equation modeling (SEM) was used to investigate direct and indirect pathways. SEM combines regression and factor analysis to allow investigation into multiple independent and multiple dependent variables simultaneously.^24,25,26^ Direct effects are associations between 2 variables. Indirect effects are associations that identify a pathway via a third variable that exists between 2 variables. Total effects include both direct and indirect effects. Cross-sectional designs can be used in SEM; however, well-fit models do not provide evidence of causation. Therefore, interpretation of results should be within the confines of the data as associations between variables. The a priori model is specified based on hypotheses and then run to determine if the hypothesized model is supported by data. Alternative models can exist and a well-fit model does not preclude other models.^24,25,26^

Before testing the hypothesized model, assumptions for SEM were tested. Descriptive statistics were run to ensure data were multivariate normal, linearly related, and at least interval scale. Stata version 18 (StataCorp) was used with maximum likelihood estimation procedure and the mlmv option to retain variables instead of allowing for listwise deletion. Statistically significant associations (*P* < .05) indicated data supported the hypothesized pathway, and structural associations were evaluated through direction and magnitude of path coefficients. Analyses used standardized β coefficients. A standardized β coefficient indicates how many SDs the dependent variable is expected to change for every 1-SD increase in the predictor variable. While typically ranging between −1 and 1, standardized coefficients can occasionally exceed this range in the presence of high collinearity or suppression effects; this does not automatically indicate an invalid model.^24^ Multiple fit statistics were investigated to identify model fit as the χ^2^ statistic is sensitive to sample size. Good fit was defined as root mean square error of approximation (RMSEA) less than 0.05, and comparative fit index (CFI) greater than 0.9.^27^ SEM requires a higher sample size to ensure stable estimates, with a recommendation of 20:1 (subject to variables) to ensure 80% power without oversaturating the model. Therefore, the sample size of 15 190 US census tracts is sufficient for estimation of the hypothesized model. All analyses were conducted from November 2023 to January 2026.

## Results

The final sample consisted of 15 190 US census tracts across 157 counties, within 50 states including Washington, DC. The mean (SD) diabetes prevalence across census tracts was 11.8% (4.9%), and a mean (SD) 26.7% (31.4%) of the population was Black or African American across census tracts. The mean (SD) census tract HOLC score was 2.96 (0.79), which is just below the score for declining neighborhoods. Mean, minimum, and maximum values for each are presented in Table 1. Pairwise correlations (Pearson *r*) between diabetes prevalence, SREI, HOLC scores, and the proportion of a census tract that reported African American or Black race are presented in Table 2.

**Table 1.** Summary Characteristics of Census Tracts in Analytic Sample^a^

Characteristic	Mean (SD) within census tract	Minimum	Maximum
Diabetes prevalence, %	11.8 (4.9)	0.9	36.1
SREI^b^	0.28 (1.31)	−4.28	4.80
Historic residential redlining (HOLC grade)	2.95 (0.80)	1	4
Proportion African American or Black race in census tract, %	26.7 (31.4)	0	100

Abbreviations: HOLC, Home Owners’ Loan Corporation; SREI, structural racism effect index.

^a^ All measures are at the census-tract level and therefore represent mean or proportion across census tracts.

^b^ Measured using a summary score of 9 domains: built environment, criminal justice, education, employment, housing, income and/or poverty, social cohesion, transportation, and wealth.

**Table 2.** Pairwise Correlations for Diabetes Prevalence, Structural Racism Effect Index (SREI), Home Owners’ Loan Corporation (HOLC) Scores, and Proportion of Census Tract Reporting African American or Black Race

Measure	Diabetes prevalence	SREI^a^	HOLC Grade	Proportion African American or Black race
Diabetes prevalence	1.0	NA	NA	NA
SREI^a^	0.79^b^	1.0	NA	NA
Historic residential redlining (HOLC grade)	0.24^b^	0.28^b^	1.0	NA
Proportion African American or Black race	0.71^b^	0.61^b^	0.20^b^	1.0

Abbreviation: NA, not applicable.

^a^ Measured using a summary score of 9 domains: built environment, criminal justice, education, employment, housing, income and/or poverty, social cohesion, transportation, and wealth.

^b^ Indicates statistically significant at *P* < .05 level for pairwise correlations (Pearson *r*).

Table 3 provides estimates and measures of variance for the direct, indirect, and total effects of the SEM model. Historic redlining (β = 0.02; 95% CI, −0.05 to 0.08; *P* = .001), contemporary structural racism (β = 0.56; 95% CI, 0.51 to 0.61; *P* < .001), and proportion of the census tract reporting Black race (β = 0.37; 95% CI, 0.18 to 0.55; *P* < .001) were directly associated with higher diabetes prevalence. Historic redlining was indirectly associated with higher diabetes prevalence via the pathway of contemporary structural racism (β = 0.17; 95% CI, 0.15 to 0.20; *P* < .001). Black race was indirectly associated with higher diabetes prevalence through both more exposure to historic redlining (β = 0.18; 95% CI, 0.14 to 0.22; *P* < .001) and higher levels of contemporary structural racism (β = 0.58; 95% CI, 0.52 to 0.63; *P* < .001). The model was well fit with χ^2^_6_ = 20 152.44, *P* < .001, RMSEA = 0.00, and CFI = 1.00.

**Table 3.** Standardized Direct, Indirect, and Total Associations for the Association Between Race, Historic Redlining, Contemporary Structural Racism, and Diabetes Prevalence^a^

Association	Direct effect (SE)	Indirect effect (SE)	Total effect (SE)
Diabetes prevalence			
Contemporary racism	0.56 (0.02)^b^	NA	0.56 (0.02)^b^
Historic redlining	0.02 (0.03)^c^	0.10 (0.03)^b^	0.11 (0.04)^b^
African American or Black race	0.37 (0.09)^b^	0.34 (0.08)^b^	0.71 (0.10)^b^
Contemporary racism			
Historic redlining	0.17 (0.01)^b^	NA	0.17 (0.01)^b^
African American or Black race	0.58 (0.03)^b^	0.03 (0.01)^b^	0.61 (0.03)^b^
Historic redlining			
African American or Black race	0.18 (0.02)^b^	NA	0.18 (0.02)^b^

Abbreviation: NA, not applicable.

^a^ Structural equation modeling (SEM) with standardized estimates were used to investigate associations. Significant SEM direct effects indicate direct association between variables. For example, higher levels of historic redlining are associated with higher diabetes prevalence. Significant SEM indirect effects indicate pathways through which variables are associated with each other. For example, higher historic redlining was associated with diabetes prevalence through contemporary structural racism. No additional adjustment was included beyond variables in the model.

^b^ Indicates *P* < .001.

^c^ Indicates *P* < .01.

**Figure 1.**
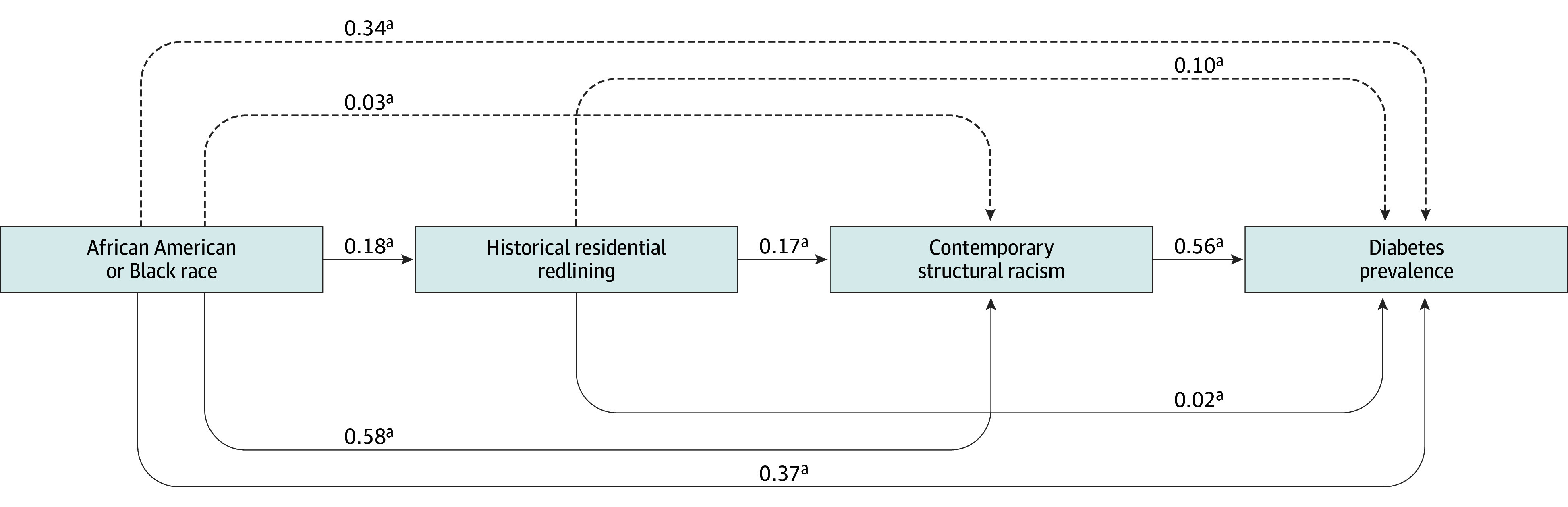
Final Model of Association Between Race, Historic and Contemporary Measures of Structural Racism, and Diabetes Prevalence in the US Structural equation modeling with standardized estimates were used to investigate associations. Standardized values have a mean (SD) of 0 (1) and allow for comparison between values to identify relative strength and direction. Solid lines show direct associations (where 2 variables are associated with each other) and dotted lines show indirect associations (where 2 variables are associated with each other via a third variable). Indirect associations indicate pathways through which variables are associated with each other. For example, higher historic redlining was associated with diabetes prevalence through contemporary structural racism. Measures of variance for each association shown in Table 3 and Figure 2. ^a^Indicates associations are significant at *P* < .001.

**Figure 2.**
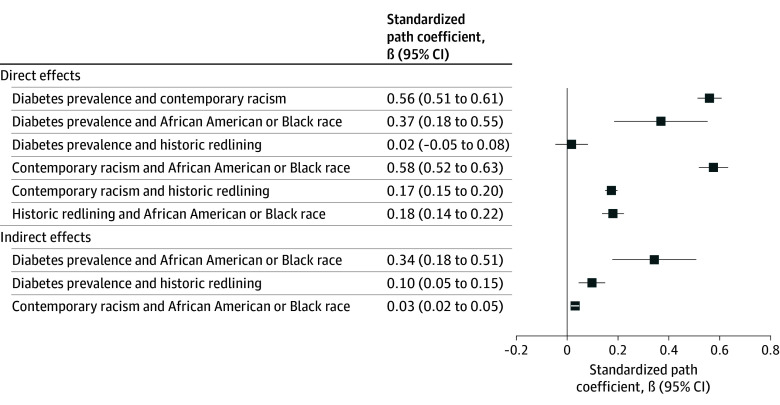
Forest Plot of Direct and Indirect Associations Between Race, Historic and Contemporary Measures of Structural Racism, and Diabetes Prevalence in the US Structural equation modeling with standardized estimates was used to create standardized estimates.

Figures 1 and 2 illustrate the direct and indirect associations in the tested model. Within this model, the strongest association was the one between African American or Black race and contemporary structural racism (β = 0.58). The second strongest was contemporary structural racism and diabetes prevalence (β = 0.56). These were each quite a bit stronger than the associations between historic residential redlining and contemporary structural racism (β = 0.17) or diabetes prevalence (β = 0.02), suggesting the importance of contemporary structural racism in understanding the overarching association of race, historic and contemporary measures of structural racism, and diabetes prevalence in the US.

## Discussion

Using US census tract-level data, this cross-sectional study provides evidence that both historic and contemporary measures of structural racism are directly associated with higher diabetes prevalence. Historic residential redlining was indirectly associated with higher diabetes prevalence via contemporary structural racism, and African American or Black race was indirectly associated with higher diabetes prevalence through both more exposure to historic redlining and higher levels of contemporary structural racism. In addition, contemporary structural racism showed a stronger association for the pathway through which Black race was associated with higher diabetes prevalence.

These findings align with a growing body of literature documenting the influence of structural racism on chronic disease outcomes. Prior studies show that racially and ethnically minoritized groups, particularly African American individuals, experience a disproportionate burden of diabetes, often driven by structural inequities in income, education, health care access, and environmental conditions.^28,29,30^ A recent national analysis similarly reported that historic redlining was directly and indirectly associated with higher diabetes prevalence, highlighting the long-lasting imprint of discriminatory housing policies.^17^ A cross-sectional study conducted in Durham County, North Carolina, found that higher burden of structural racism indicators, such as a lower percentage of White individuals, higher reported violent crimes, and higher levels of poverty, were associated with greater neighborhood prevalence of chronic disease, including diabetes.^31^

Unlike many previous studies that focus either on historic or contemporary forms of racism in isolation, our study simultaneously investigated the direct and indirect associations of both historic redlining and contemporary structural racism, using a novel, multidomain measure (SREI). This dual focus supports our hypotheses that past discriminatory policies contribute to present-day inequities in social determinants of health, which ultimately are associated with present day disparities in diabetes prevalence. The finding that contemporary structural racism is the strongest mediator between Black race and diabetes prevalence suggests that structural inequities are not only historically rooted but are actively maintained through current policies and systems. It is important to note that remaining direct effects between race and diabetes prevalence is not a result of race itself, but additional factors not captured in the historic residential redlining and SREI index included in this study. This may include additional historic structural factors, such as the impact of Jim Crow laws on employment and educational opportunities, unequal access to health care due to segregated hospitals, disproportionate environmental exposures, or unequal access to insurance. There may also be additional aspects of contemporary structural racism that are not captured in the SREI index. Given the limited data captured at a national level that can be used to understand structural factors, additional variables that can explain the remaining direct effects should not limit action that can be taken using results from the current analysis as these findings have significant implications across policy, clinical practice, and research.

From a policy perspective, efforts to reduce diabetes disparities must move beyond individual-level interventions and confront the broader structural inequities that shape health. One option for structural change is policy efforts to expand the reach and equity of social support programs such as the Supplemental Nutrition Assistance Program, Temporary Assistance for Needy Families, and the Low-Income Home Energy Assistance Program. Similarly, expanding Medicaid at the state level can help remove financial barriers to care for underserved populations. Investment in integrated care models that address both medical and social needs, such as medical-legal partnerships and housing support services,^32,33^ is also essential to mitigate the structural inequities that contribute to diabetes risk.

In terms of clinical practice, health care practitioners must be equipped with the skills and knowledge to understand the upstream drivers of diabetes disparities impacting their patients. This includes training in structural competency and culturally responsive care.^34^ Expanding care teams to include community health workers, mental health professionals, and social need navigators can further help patients navigate barriers beyond the clinical setting and offer practitioners a way to ensure individuals with social needs receive linkages to these services.^35^ Moreover, clinical practice should shift from merely screening for social risks to actively resolving patient-defined social needs that interfere with diabetes self-management and outcomes through medical-social partnerships.^36^

Future research should prioritize the design and evaluation of multilevel interventions that integrate neighborhood-level structural factors with individual health outcomes. Studies should explore collaborative models that bring together health care systems, public health agencies, and community-based organizations to deliver sustainable diabetes prevention programs. An example of this is deploying the Diabetes Prevention Program through trusted community channels in underserved areas.^37^ Innovative methods are needed to examine the complex interplay of structural drivers, assess mediating pathways, and capture the cumulative and life-course effects of systemic racism on diabetes risk.^35^

### Limitations

Though it uses national data and incorporates both historic and contemporary measures of structural racism, this study has several limitations that should be noted. First, the cross-sectional design limits causal inference. Pathways were modeled statistically; however, temporal associations cannot be confirmed, and additional work using longitudinal data are needed to confirm the causal nature of these pathways. Second, since historic redlining primarily occurred in urban areas, results may not be generalizable to rural regions that lack this specific history but may still experience structural racism through other mechanisms. Third, although the SREI captures multiple domains of structural racism, it may not fully capture all contemporary forms of racism or their intersections.

## Conclusions

This study found that both historic redlining and contemporary structural racism are significantly associated with higher diabetes prevalence at the census-tract level in the US. Importantly, contemporary structural racism serves as a powerful pathway through which historic injustices are associated with current diabetes outcomes. These findings reinforce the need for targeted policies that expand the reach and equity of social support programs, equip health care systems with the skills and partnerships to address upstream drivers of diabetes disparities, and prioritize the design and evaluation of multilevel interventions that address these drivers.

## Notes

Supplement. Data Sharing Statement
