# Supplementary material for: Race, Structural Racism, and Prevalence of Diabetes in US Neighborhoods
Source: JAMA Netw Open. 2026 Apr 6;9(4):e265122. doi: 10.1001/jamanetworkopen.2026.5122 (PMC13054619; doi:10.1001/jamanetworkopen.2026.5122)
Supplement: Supplement. — Data Sharing Statement [file jamanetwopen-e265122-s001.pdf]

Data Sharing Statement

Egede. Race, Structural Racism, and Prevalence of Diabetes in US Neighborhoods. *JAMA Netw Open*. Published April 06, 2026. doi:10.1001/jamanetworkopen.2026.5122

Data
Data available: No

Additional Information
Explanation for why data not available: Data and Resource Availability Statement: The analytic dataset was created by combining census tract level data across multiple sources including CDC PLACES (https://www.cdc.gov/places/index.html), Mapping Inequality Project (https://dsl.richmond.edu/panorama/redlining/), American Community Survey data (https://www.census.gov/programs-surveys/acs/data.html), and Dyer et al's Structural Racism Effect Index data (https://doi.org/10.1377/hlthaff.2023.00659).
